# Adverse childhood experiences, traumatic events, and mental health among adults at two outpatient psychiatric facilities in Johannesburg, South Africa: a cross-sectional analysis

**DOI:** 10.1186/s12888-023-05085-0

**Published:** 2023-08-10

**Authors:** William Byansi, Michael Galvin, Lesley Chiwaye, Zoleka Luvuno, Andrew W. Kim, Radhika Sundararajan, Alexander C. Tsai, Aneesa Moolla

**Affiliations:** 1 School of Social Work, Boston College, 140 Commonwealth Avenue, Chestnut Hill, MA 02467 USA; 2 Harvard T.H. Chan School of Public Health, Boston, MA USA; 3 Boston Medical Center, Department of Psychiatry, Boston, MA USA; 4 Faculty of Health Sciences, Health Economics and Epidemiology Research Office, University of the Witwatersrand, Johannesburg, South Africa; 5 Department of Anthropology, University of California, Berkeley, United States; 6 Weill Cornell Center for Global Health, New York City, New York United States; 7 Department of Emergency Medicine, Weill Cornell Medicine, New York City, New York United States; 8 Center for Global Health and Mongan Institute, Massachusetts General Hospital, Boston, MA USA; 9 Harvard Medical School, Boston, MA USA; 10 Department of Epidemiology, Harvard T.H. Chan School of Public Health, Boston, MA USA

**Keywords:** Adverse childhood experiences, Anxiety, Depression, Interpersonal violence, Mental illness, Stress, Sub-saharan Africa, Post-traumatic stress, Psychiatric disorders

## Abstract

**Background:**

Adverse childhood experiences and adult trauma, including sexual abuse, physical abuse, neglect, and interpersonal violence, are highly prevalent in low-resource settings and associated with adverse psychological outcomes. However, there is limited focus on the impact of ACEs and trauma on mental health in sub-Saharan Africa. Therefore, this study examines the impact of traumatic events and ACEs on depression, anxiety, and stress scores among outpatients receiving psychiatric care at two public mental health treatment facilities in Johannesburg, South Africa.

**Methods:**

A sample of 309 participants were recruited between January and June 2022 at Helen Joseph Hospital and Alexandra 18th Avenue Clinic. Participants completed screening measures for mental health outcomes, including the 9-item Patient Health Questionnaire (PHQ-9), the 7-item General Anxiety Disorder scale (GAD-7) and the 10-item Perceived Stress Scale. We fitted modified Poisson and linear regression models to estimate the impact of ACEs and adult experiences of trauma on depression, anxiety, and stress scale scores.

**Results:**

47.57% (n = 147) of participants screened positive for anxiety, 44.66% (n = 138) for depression, and 17% (n = 54) for severe stress. More females screened positive for anxiety (65.31%), depression (65.94%), and stress (77.78%). Each ACE was associated with a 12% increased risk of depression, a 10% increased risk of anxiety, and a 17% increased risk of stress. In separately estimated models, each additional traumatic event during adulthood was associated with a 16% increased risk for depression, an 8% increased risk of anxiety, and a 26% increased risk of stress. Across all models, being male and self-reported physical health were consistently associated with a reduced risk for depression, anxiety, and stress.

**Conclusions:**

ACEs and experiences of traumatic events as adults were associated with significantly increased risks of anxiety, depression, and severe stress. Given high exposure to ACEs and trauma and the associated impact on the mental health of individuals, families, and communities, there is a need to strengthen and scale innovative combination interventions that address multiple stressors impacting people in low-resource settings.

**Supplementary Information:**

The online version contains supplementary material available at 10.1186/s12888-023-05085-0.

## Background

Mental, neurological, and substance use disorders represent 9 of the 20 leading causes of disability-adjusted life years (DALYs) worldwide [1, 2]. Depression and anxiety constitute a substantial proportion of the global disease burden and are the second most common cause of disability [3, 4]. A large segment of international mental health services research is currently focused on how best to close mental health treatment gaps in developing countries to improve sustainability, cultural relevance, and quality of services. This increased importance of non-communicable diseases such as anxiety and depressive disorders presents a particular challenge for low-income countries, where infectious diseases and malnutrition are still rife and where only a low percentage of national budgets are allocated to health services. In South Africa, mental illness is a significant issue with high prevalence – estimated at more than 30% lifetime risk [5, 6]. If left untreated, these disorders can be chronic, resulting in adverse effects on morbidity and mortality [7, 8].

South Africa is characterized by high levels of poverty, interpersonal violence, and a societal history of trauma [9], all of which are associated with a higher risk of mental health disorders [10]. Studies in southern and eastern Africa have shown strong relationships between adverse childhood experiences (ACEs) – such as sexual abuse, physical abuse, and neglect – and psychological outcomes during adulthood [11–15]. In one study of 2,427 young men, childhood trauma was shown to correlate strongly with depressive symptoms in adulthood [12]. As South Africa is already burdened by stressors such as poverty, violence, and HIV, studies show that traumatic experiences are not limited to childhood and indicate significantly increased vulnerabilities to stress, depression, and anxiety symptoms [16–20]. In this setting, while some studies have looked at the impact of ACEs and trauma on mental health among children, pregnant women, and people living with HIV, no studies have focused on outpatients receiving psychiatric care. This group is highly vulnerable to violence, stigma, trauma and injury [21]. More recently, the impacts of the COVID-19 pandemic strongly affected the South African population, and the pandemic’s socioeconomic aftereffects exacerbated already high levels of poverty, violence, and stress in local communities [22]. To improve clinical outcomes and ensure that individuals receive the care they need, it is important to examine the prevalence of mental health challenges and their relationship to traumatic experiences in both childhood and adulthood among individuals receiving psychiatric care.

There is limited attention to ACEs and adult trauma, especially in sub-Saharan Africa, a region heavily impacted by poverty, food and water insecurity, violence and communicable and non-communicable diseases. Additionally, trauma in childhood and adulthood is highly prevalent and poses significant consequences to the mental health of individuals, households, and communities [12, 23]. Many patients who experience mental illness due to ACEs, trauma, or other adverse life events often benefit significantly from evidence-based trauma informed psychosocial support in the form of psychotherapy or counseling [24, 25]. However, few public psychiatric facilities in South Africa currently provide significant psychosocial treatment options, instead relying on brief visits with psychiatrists who prescribe medication. Therefore, this study aimed to determine the association between ACEs and adult trauma, and depression, anxiety, and stress among outpatients at two psychiatric facilities in Johannesburg, South Africa, and to propose solutions for the future of public psychiatric care in the country.

## Methodology

### Sample

Data for this cross-sectional study were collected between January and July 2022 at two public psychiatric facilities in Johannesburg, South Africa – Helen Joseph Hospital and the Alexandra 18th Avenue Clinic. This study assessed the perceptions and experiences of mental illness and treatment among psychiatric patients at these locations. A convenience sampling technique was used to recruit participants receiving outpatient psychiatric care at two hospitals in Johannesburg, South Africa (n = 309). All patients who identified as being of Black/African descent, aged 18 years or above, and willing/able to provide written informed consent were eligible to participate in the study. Individuals who did not meet the inclusion criteria or self-reported having a severe mental illness or disability that would prevent them from participating in the survey were excluded from the study.

### Procedures

The current study was granted ethical approval from the University of the Witwatersrand (M210815) and the Johannesburg Health District (GP_202111_059). Potential participants were approached by trained research assistants at the two hospitals. Additionally, approved flyers describing the study were given to primary healthcare providers to share with their patients. The flyers gave a brief description of the study, and the days, times, and locations at the hospital where the researchers would be conducting the surveys. After written informed consent was obtained, each participant was interviewed by a trained research assistant (RA). The RAs were fluent in isiZulu, isiXhosa, and English. The interviewer administered survey lasted about 40 min and was completed in the language preferred by the participant. To ensure confidentiality, the interviews were conducted in-person at a private location at the hospital. All potential participants were screened for eligibility in-person. All eligible participants signed and received a copy of the consent form to keep for their records. Survey participants received remuneration in the amount of 100 South African Rand (approximately $7 US at the time the study was conducted).

### Measures

Participants were asked to self-report their sex, age, family psychiatric history, personal income, marital status, education level, and the number of people in the household. Sex was measured as a binary variable (male or female). Age (range 18–71 years), personal income (0–80,000 ZAR), number of people in the household (1–21 adults), and number of children in the household (0–7 children) were measured as continuous variables. Marital status (married, single, other) and educational level (primary, high school, and college/university) were measured as categorical variables. Family psychiatric history was measured as a binary variable (yes/no), indicating whether any family member had experienced any mental health problems. HIV status was a self-reported binary variable indicating whether the participants knew their HIV sero-status (negative/positive).

Self-rated physical health was assessed through a single question that asked the participant to rate their overall physical health on a 4-point Likert scale ranging from 1 = very good to 4 = very bad. For this analysis, scores were reverse coded for higher scores to indicate better physical health.

Adverse childhood experiences (ACEs) were measured with the 10 items version of the Adverse Childhood Experiences–International Questionnaire [26]: emotional and/or physical neglect; emotional, sexual, and/or physical abuse; being bullied; living in the same household as someone engaged in substance use and/or someone with a mental disorder; having a household member being incarcerated; witnessing domestic violence in the household; experiencing parental loss and/or parental divorce or separation; witnessing interpersonal violence in the community; and being exposed to collective violence, such as war, terrorism, organized violence, or political violence. In this study, all items on the instrument were binary variables, with any experience of the event categorized as 1 and no experience of the event categorized as 0. These 10 binary variables were summed to calculate the cumulative number of ACEs (Cronbach’s alpha = 0.67).

Traumatic events were measured using an adapted 12-item index [27] assessing experiences of potentially traumatic events (or social disorder, similar to ACEs) during adulthood, i.e., occurring since the participant was 18 years of age: lack of medical care, homelessness, imprisonment, family separation, injury, rape, difficult pregnancy, murder, natural disaster, victim of a violent attack, domestic violence, and serious automobile accident. A high index score indicates a larger number of traumatic events experienced by the participant.

Stress was measured using the 10-item Perceived Stress Scale (PSS) [28]. Participants were asked to assess their stressful life experiences in the past month. The scores range from 0 to 40 (Cronbach’s alpha = 0.86). The scale has been previously used in South Africa [28]. A score of 27 [29] and above was considered a positive screen for severe stress. In this study, the scale had an acceptable internal consistency (Cronbach’s alpha = 0.86).

Anxiety was assessed using the Generalized Anxiety Disorder screen (GAD-7). The measure consists of 7 Likert scale items that have been validated for assessing GAD in clinical and research environments in different cultural settings [30]. The 7-item measure assessed the severity of symptoms according to reported responses with a maximum possible score of 21 (Spitzer et al., 2006). Participants were asked about the symptoms experienced in the last two weeks, such as “feeling nervous, anxious or on edge” and “not being able to stop or control worrying”. A score of 8 and above was considered a positive screen for anxiety. In this study, the scale had an acceptable internal consistency (Cronbach’s alpha = 0.84).

Depression symptom severity was measured using the Patient Health Questionnaire [30–32]. The scale consists of nine items scored on a 3-point Likert-type scale ranging from 0 (not at all) to 3 (nearly every day). We used a cut-off of 10, which is generally accepted as a positive screen for depressive symptoms. In this study, the measure presented a good internal consistency (Cronbach’s alpha = 0.84).

### Statistical analysis

Descriptive analyses were run to describe the sample and characteristics associated with depression, anxiety, and stress. The prevalence of the three disorders was determined as the number of people in the sample with the characteristic of interest (positive for the disorder) divided by the total number of people in the sample as assessed using screening tools for depression, anxiety, and stress. For each outcome, we fitted four different models. We used the modified Poisson regression models [33] to estimate the relative risk of the outcomes associated with the covariates, alternately specifying ACEs (Model 1) or traumatic events during adulthood (Model 2) as the primary explanatory variables of interest. Each outcome variable was scored with 1 representing the presence of probable anxiety, depression, and/or stress, and zero representing the absence of anxiety, depression, and/or stress. Based on previous scientific research, the following covariates were identified and selected for inclusion: sex, age, family psychiatric history, personal income, marital status, education level, and the number of people in the household [34]. The two primary explanatory variables, ACEs and adult trauma, were included in separate regression models to address multicollinearity between adverse childhood experiences and traumatic events. As a sensitivity analysis, we used linear regression models to estimate the association between ACEs and mental health outcomes (model 3), and between traumatic events and mental health outcomes (Model 4), specifying depression, anxiety, and stress symptom severity as continuous variables (rather than probable depression, anxiety, and stress as binary outcome variables). We conducted this supplementary analysis given that mental health is generally understood to range on a continuum rather than a binary scale of disorder/no-disorder. All models were fitted in Stata software version 15.

## Results

### Characteristics of study participants

Of the 309 participants in this study, 56% and 44% were females and males, respectively (Table 1). On average, participants were 38 years of age (standard deviation [SD] = 12.50), lived in families with three people per household (SD = 2.4), and had about two children below 18 years of age (SD = 1.57). A majority of participants lived in townships surrounding Johannesburg. 68% (68%) of the respondents were single, 17% were married, and 15% reported other marital statuses. Nearly half of participants (45%) reported having attained at least a high school level education, and had a family member with a mental illness (43%). 40% of patients reported having received a diagnosis of depression by clinical staff, 13% reported a diagnosis of anxiety, 38% reported a diagnosis of bipolar disorder, and 9% reported a diagnosis of a psychotic disorder. Participants reported moderately high levels of depression (mean [M] = 9.83, SD = 6.2), anxiety (M = 7.90, SD = 4.8), and stress (M = 20.28, SD = 6.52). Additionally, participants reported exposure to an average of 3.21 ACEs (SD = 2.21) and 2.50 traumatic events as adults (SD = 1.74).

**Table 1:** Characteristics of Study Participants (N = 309)

Variable	N (%) or Mean (SD)
Age in years	38.52 (12.50)
Sex	
Male	137 (44%)
Female	172 (56%)
Adverse childhood experiences	3.21 (2.21)
Traumatic events during adulthood	2.50 (1.74)
History of psychiatric illness in the family	132 (43%)
Number of people in the household	3.87 (2.40)
Number of children in the family	1.40 (1.57)
Monthly income, ZAR	5897 (9173)
Marital status	
Married	52 (17%)
Single	210 (68%)
Widowed/divorced/separated	47 (15%)
Formal education	
Completed primary school	107 (35%)
Completed high school	140 (45%)
Some college/university	62 (20%)
Self-rated physical health	1.85 (0.45)
HIV-positive, self-reported	52 (17%)
Prior diagnosis given by hospital staff, self-reported	
Depression	124 (40%)
Anxiety	40 (13%)
Bipolar	117 (38%)
Psychosis	28 (9%)

### Prevalence of anxiety, depression, and stress

In total, 147 (48%) participants screened positive for anxiety (Table 2). Of these, more women (65%) than men (35%) screened positive for anxiety (*χ*^*2*^ = 10.56, p = 001). In addition, 138 (45%) of all participants screened positive for depression. Similar to anxiety, more women (66%) compared to men (34%) screened positive for depression (*χ*^*2*^ = 10.68, p = 001). Fifty-four (17%) of the participants screened positive for stress, with more women (78%) than men (22%) evidencing severe stress (*χ*^*2*^ = 12.97, p < .001).

**Table 2:** Prevalence of Depression, Anxiety, and Stress, Stratified by Sex (N = 309)

	Overall	Women	Men	
	n (%)	n (%)	n (%)	* χ* ^*2*^
**Depression**				
Positive Screen	138 (45%)	91 (66%)	47 (34%)	10.68***
Mean Score (SD)	9.83 (6.2)	11.19 (6.5)	8.14 (5.2)	
**Anxiety**				
Positive Screen	147 (48%)	96 (65%)	51 (35%)	10.56***
Mean Score (SD)	7.90 (4.8)	8.78 (4.9)	6.80 (4.3)	
**Stress**				
Positive Screen	54 (17%)	42 (78%)	12 (22%)	12.97***
Mean Score (SD)	20.28 (6.5)	21.60 (6.5)	18.63 (6.1)	

*Footnote: Depression was measured using the 9-item Patient Health Questionnaire, with a score ≥10 considered a positive screen for clinically significant symptoms of depression. Anxiety was measured using the 7-item Generalized Anxiety Disorder scale, with a score ≥8 considered a positive screen for clinically significant symptoms of anxiety. Stress was measured using the 10-item Perceived Stress Scale, with a score ≥26 considered to be indicative of severe stress.*

*p ≤ .05; **p ≤ .01; ***p ≤ .001

### ACEs, traumatic events, and depression

The results of modified Poisson and linear regression models are presented in Table 3. We fitted separate models to estimate the association between ACEs and depression, and to estimate the association between traumatic events and depression. In model 1, results indicate that each ACE was associated with a 12% increased risk of depression (adjusted relative risk [ARR] = 1.12; 95% confidence interval [CI], 1.06–1.18; *P < .001*). An increase in self-reported physical health reduced the risk of depression by 51% (ARR = 0.49; 95% CI, 0.40–0.61; *P < .001*). Results in model 2 indicate that each traumatic event was associated with a 16% increased risk for depression (ARR = 1.16; 95% CI, 1.09–1.23; *P < .001*). Additionally, self-reported physical health reduced the risk of depression by 50% (ARR = 0.50; 95% CI, 0.40–0.63; *P < .001*).

**Table 3:** Correlates of Probable Depression and Depression Symptom Severity (N = 309)

	Adjusted relative risk ratio (95% confidence interval (CI), p-value		Unstandardized regression coefficient (95% CI), p-value	
	**Model 1**	**Model 2**	**Model 3**	**Model 4**
**Variable**				
ACEs, per point	**1.12 (1.06, 1.18), < 0.001**		**0.51 (0.23, 0.80), < 0.001**	
Traumatic events, per point		**1.16 (1.09, 1.23), < 0.001**		**0.82 (0.47, 1.17), < 0.001**
Age, per year	0.99 (0.99, 1.01), 0.89	0.99 (0.98, 1.01), 0.31	-0.05 (-0.12, 0.01), 0.12	**-0.08 (-0.15, -0.02), 0.01**
Male sex (ref: female sex)	0.82 (0.62, 1.09), 0.18	**0.75 (0.57, 0.98), 0.04**	**-1.98 (-3.32, -0.64), 0.004**	**-2.34 (-3.65, -1.03), < 0.001**
History of psychiatric illness in the family (ref: No)	0.86 (0.67, 1.12), 0.24	0.92 (0.73, 1.16), 0.48	0.39 (-0.90, 1.67), 0.56	0.51 (-0.72, 1.75), 0.41
People in the household, per person	0.99 (0.95, 1.03), 0.57	0.98 (0.94, 1.03), 0.48	-0.21 (-0.46, 0.04), 0.09	-**0.25 (-0.50, -0.003), 0.047**
Children in the family, per child	0.96 (0.87, 1.05), 0.38	0.96 (0.87, 1.05), 0.37	-0.23 (-0.70, 0.24), 0.34	-0.19 (-0.65, 0.28), 0.43
Monthly income, per ZAR	0.99 (0.99, 1.00), 0.37	0.99 (0.99, 1.00), 0.37	-0.00 (-0.00, 0.004), 0.49	-0.00 (-0.00, 0.00), 0.57
Marital status				
Married	Ref	Ref	Ref	Ref
Single	1.23 (0.83, 1.84), 0.31	1.21 (0.80, 1.82), 0.36	1.14 (-0.79, 3.06), 0.25	0.97 (-0.93, 2.86), 0.32
Widowed/divorced /separated	1.14 (0.74, 1.77), 0.55	1.06 (0.67, 1.67), 0.80	0.61 (-1.60, 2.81), 0.59	0.26 (-1.91, 2.43), 0.82
Education				
Completed primary school	Ref	Ref	Ref	Ref
Completed high school	**1.55 (1.15, 2.09), < 0.001**	**1.51 (1.12, 2.02), 0.01**	**2.06 (0.65, 3.46), < 0.01**	**1.84 (0.46, 3.22), 0.01**
Some college/ university	**1.50 (1.05, 2.15), 0.025**	**1.44 (1.01, 2.05), 0.04**	1.80 (-0.04, 3.64), 0.06	1.53 (-0.27, 3.34), 0.10
Self-rated physical health, per point	**0.49 (0.40, 0.61), < 0.001**	**0.50 (0.40, 0.63), < 0.001**	**-5.11 (-6.52, -3.70), < 0.001**	**-4.90 (-6.30, -3.51), < 0.001**
HIV positive	0.77 (0.55, 1.08), 0.13	0.74 (0.53, 1.03), 0.08	-0.97 (-2.62, 0.68), 0.25	-1.12 (-2.73, 0.51), 0.18

Specifying depression symptom severity as a continuous outcome yielded estimates that were largely consistent with the estimated relative risks. In model 3, depression symptom severity was associated with ACEs (*b* = 0.51 per ACE, 95% CI = 0.23–0.80, *P < .001*), male sex (*b* = -1.98, 95% CI = -3.32 to -0.64, *P < .001*), and self-rated physical health (*b* = -5.11 per point on the self-rated health scale, 95% CI = -6.52 to -3.70, *P ≤ .001*). In model 4, depression symptom severity was associated with traumatic events during adulthood (*b* = 0.82 per traumatic event, 95% CI = 0.47–1.17, *P < .001*), male sex (*b* = -2.34, 95% CI = -3.65 to -1.03, *P < .001*), age (*b* = -0.08 per year, 95% CI = -0.15 to -0.02, *P = .01*), and self-rated physical health (*b* = -4.90 per point on the self-rated health scale, 95% CI = -6.30 to -3.51, *P < .001*).

### ACEs, traumatic events, and anxiety

Modified Poisson and linear regression analysis indicated that ACEs, traumatic events, self-rated physical health, education levels, and family psychiatric history were associated with probable anxiety and anxiety symptom severity (Table 4). In model 1, results indicate that each ACE was associated with a 10% increased risk of probable anxiety (adjusted relative risk [ARR] = 1.10; 95% confidence interval [CI], 1.04–1.15; *P < .001*). Each point on the self-reported physical health scale, however, was associated with a 46% reduced risk for probable anxiety (ARR = 0.54; 95% CI, 0.44–0.67; *P < .001*). Similar to ACEs, in model 2, each traumatic event was associated with an 8% increased risk of probable anxiety (ARR = 1.08; 95% CI, 1.02–1.15; *P = .01*). On the other hand, there was a 33% reduced risk of probable anxiety associated with male sex (ARR = 0.77; 95% CI, 0.59–0.99; *P = .05*) and a 46% reduced risk associated with each point on the self-reported physical health scale (ARR = 0.54; 95% CI, 0.44–0.66; *P < .001*).

**Table 4:** Correlates of Probable Anxiety and Anxiety Symptom Severity (N = 309)

	Adjusted relative risk ratio (95% confidence interval (CI), p-value		Unstandardized regression coefficient (95% CI), p-value	
	**Model 1**	**Model 2**	**Model 3**	**Model 4**
**Variable**				
ACEs, per point	**1.10 (1.04, 1.15), < 0.001**		**0.52 (0.31, 0.74], < 0.001**	
Traumatic events, per point		**1.08 (1.02, 1.15), 0.01**		**0.60 (0.33, 0.87), < 0.001**
Age, per year	1.00 (0.99, 1.01), 0.70	0.99 (0.97, 1.01), 0.74	-0.02 (-0.07, 0.02), 0.35	**-0.05 (-0.10, -0.00), 0.04**
Male sex (ref: female sex)	0.83 (0.64, 1.09), 0.19	**0.77 (0.59, 0.99), 0.05**	**-1.16 (-2.20, -0.13), 0.03**	**-1.53 (-2.56, -0.50), 0.004**
History of psychiatric illness in family (ref: No)	1.21 (0.95, 1.54), 0.12	**1.32 (1.05, 1.65), 0.02**	0.54 (-0.46, 1.53), 0.29	0.85 (-0.12, 1.82), 0.09
People in the household, per person	0.98 (0.94, 1.02), 0.32	0.97 (0.93, 1.02), 0.23	-0.05 (-0.25, 0.14), 0.59	-0.09 (-0.28, 0.11), 0.37
Children in the family, per child	0.95 (0.86, 1.04), 0.24	0.94 (0.86, 1.04), 0.23	-0.17 (-0.53, 0.20), 0.37	-0.14 (-0.50, 0.23), 0.47
Monthly income, per ZAR	0.99 (0.99, 1.00), 0.62	0.99 (0.99, 1.00), 0.62	-0.00 (-0.00, 0.00), 0.59	-9.36 (-0.00, 0.00), 0.73
Marital status				
Married	Ref	Ref	Ref	Ref
Single	1.12 (0.78, 1.60), 0.54	1.09 (0.76, 1.56), 0.64	1.31 (-0.17, 2.80), 0.08	1.13 (-0.36, 2.62), 0.14
Widowed/divorced /separated	0.90 (0.59, 1.37), 0.62	0.86 (0.56, 1.31), 0.47	0.22 (-1.48, 1.92), 0.79	-0.09 (-1.80, 1.62), 0.92
Education				
Completed primary school	Ref	Ref	Ref	Ref
Completed high school	**1.35 (1.02, 1.79), 0.03**	1.31 (0.99, 1.74), 0.06	**1.83 (0.75, 2.92), < 0.001**	**1.61 (0.52, 2.70), 0.004**
Some college/ university	1.26 (0.90, 1.78), 0.17	1.22 (0.88, 1.69), 0.24	**1.66 (0.24, 3.08), 0.02**	1.38 (-0.04, 2.80), 0.06
Self-rated physical health, per point	**0.54 (0.44, 0.67), < 0.001**	**0.54 (0.44, 0.66), < 0.001**	**-3.66 (-4.75, -2.58), < 0.001**	**-3.56 (-4.65, -2.46), < 0.001**
HIV Positive	0.84 (0.61, 1.17), 0.31	0.81 (0.58, 1.13), 0.21	-0.83 (-2.10, 0.44), 0.20	-0.96 (-2.24, 0.32), 0.14

In model 3, anxiety symptom severity was associated with ACEs (*b* = 0.52, per ACE, 95% CI = 0.31, 0.74, *P < .001*), male sex (*b* = -1.16, 95% CI = -2.20, -0.13, *P = .03*), and self-rated physical health (*b* = -3.66, per point on the self-rated health scale, 95% CI = -4.75, -2.58, *P < .001*). In model 4, anxiety symptom severity was associated with traumatic events during adulthood (*b* = 0.60, per traumatic event, 95% CI = 0.33, 0.87, *P < .001*), male sex (*b* = -1.53, 95% CI = -2.56, -0.50, *P = .004*), and self-rated physical health (*b* = -3.56, per point on the self-rated health scale, 95% CI = -4.65, -2.46, *P < .001*).

### ACEs, traumatic events, and stress

Table 5 presents separate models that estimate the association between ACEs and stress, and that estimate the association between traumatic events and stress. In model 1, the results indicate that each ACE was associated with a 17% increased risk of stress (ARR = 1.17; 95% CI, 1.06–1.28; *P < .001*). Additionally, each point on the self-rated physical health scale was associated with a 58% risk reduction in stress (ARR = 0.42; 95% CI, 0.27–0.68; *P < .001*). In model 2, each traumatic event was associated with a 26% increased risk of stress (ARR = 1.26; 95% CI, 1.12–1.42; *P < .001*). On the other hand, male sex (ARR = 0.48; 95% CI, 0.27–0.87; P = .02) and each point on the self-rated health scale (ARR = 0.46; 95% CI, 0.29–0.74; *P < .001*) were associated with a reduced risk of probable stress.

**Table 5:** Correlates of Probable Stress and Stress Symptom Severity (N = 309)

	Adjusted relative risk ratio (95% confidence interval (CI), p-value		Unstandardized regression coefficient (95% CI), p-value	
**Variable**	**Model 1**	**Model 2**	**Model 3**	**Model 4**
Traumatic events, per point	**1.17 (1.06, 1.28), < 0.001**		**0.77 (0.46, 1.08), < 0.001**	
Traumatic events		**1.26 (1.12, 1.42), < 0.001**		**1.01 (0.63, 1.40), < 0.001**
Age, per year	**0.96 (0.94, 0.99), 0.01**	**0.95 (0.92, 0.98), 0.01**	-0.02 (-0.09, 0.05), 0.49	**-0.07 (-0.14, -0.003), 0.05**
Male sex (ref: female sex)	0.59 (0.31, 1.12), 0.12	**0.48, (0.27, 0.87), 0.02**	-1.27 (-2.75, 0.21), 0.09	**-1.81 (-3.27, -0.35), 0.02**
History of psychiatric illness in the family (ref: No)	1.13 (0.69, 1.83), 0.63	1.24 (0.77, 1.98), 0.37	0.13 (-1.29, 1.55), 0.86	0.49 (-0.88, 1.86), 0.48
People in the household, per person	0.97 (0.88, 1.07), 0.54	0.97 (0.87, 1.07), 0.53	-0.08 (-0.36, 0.19), 0.55	-0.14 (-0.41, 0.14), 0.33
Children in the family, per child	1.03 (0.80, 1.32), 0.83	1.00 (0.78, 1.28), 0.98	0.09 (-0.43, 0.61), 0.73	0.14 (-0.37, 0.66), 0.59
Monthly income, per ZAR	0.99 (0.99, 1.00), 0.75	0.99 (0.99, 1.00), 0.69	-0.00 (-0.00, 0.00), 0.47	-0.00 (-0.00, 0.00), 0.61
Marital status				
Married	Ref	Ref	Ref	Ref
Single	1.01 (0.46, 2.23), 0.98	1.02 (0.46, 2.27), 0.95	**2.63 (0.50, 4.75), 0.02**	**2.36 (0.25, 4.47), 0.03**
Widowed/divorced /separated	1.48 (0.60, 3.67), 0.40	1.52 (0.61, 3.79), 0.37	2.30 (-0.12, 4.73), 0.06	1.82 (-0.60, 4.23), 0.14
Education				
Completed primary school	Ref	Ref	Ref	Ref
Completed high school	**1.91 (1.00, 3.66), 0.05**	**1.86 (1.00, 3.47), 0.05**	0.38 (-1.16, 1.93), 0.63	0.06 (-1.48, 1.59), 0.94
Some college/ university	2.01 (0.98, 4.10), 0.06	1.90 (0.95, 3.83), 0.07	1.64 (-0.38, 3.67), 0.11	1.23 (-0.78, 3.24), 0.23
Self-rated physical health, per point	**0.42 (0.27, 0.68), < 0.001**	**0.46 (0.29, 0.74), < 0.001**	**-4.45 (-5.99, -2.88), < 0.001**	**-4.22 (-5.77, -2.67), < 0.001**
HIV positive	1.16 (0.63, 2.15), 0.63	1.06 (0.63, 1.79), 0.83	0.84 (-0.97, 2.66), 0.36	0.64 (-1.16, 2.45), 0.48

In model 3, stress symptom severity was associated with ACEs (*b* = 0.77, per ACE, 95% CI = 0.46, 1.08, *P < .001*), and self-rated physical health (*b* = -4.45, per point on the self-rated health scale, 95% CI = -5.99, -2.88, *P* < .001). In model 4, stress symptom severity was associated with traumatic events during adulthood (*b* = 1.01, per traumatic event, 95% CI = 0.63, 1.40, *P < .001*), male sex (*b* = -1.81, 95% CI = -3.27, -0.35, *P = .02*), and self-rated physical health (*b* = -4.22, per point on the self-rated health scale, 95% CI = -5.77, -2.67, *P < .001*).

## Discussion

The study aimed to examine the impact of traumatic events and ACEs on depression, anxiety, and stress scores among adult outpatients receiving psychiatric care at two public mental health treatment facilities in Johannesburg, South Africa. On average, participants reported 3 ACEs and 2 adult traumatic events each, estimates that were comparable with other recent studies examining clinical populations in South Africa [35]. We found that these ACEs and experiences of traumatic events as adults were associated with increased risks of anxiety, depression and severe stress. The estimated associations were small to moderate in magnitude. For example, a one standard deviation difference in exposure to traumatic events during adulthood was associated with a 1.74 × 0.82 = 1.43 difference in depression symptom severity, 1.43/6.2 = 0.23 standard deviation units. The same exposure difference was associated with a 1.74 × 1.01 = 1.76 difference in symptoms of stress, 1.76/6.5 = 0.27 standard deviation units. Using conventional rules of thumb, these estimated associations would be considered “small” to “medium” effect sizes.

Our findings of elevated risks of poor mental health outcomes associated with ACEs are consistent with previous research indicating that ACEs have cumulative negative impacts on physical and mental health during adulthood [23, 36]. In other parts of sub-Saharan Africa, longitudinal studies indicate that youths who have experienced trauma frequently show increased aggression, anger, depression, hopelessness, withdrawal, and social isolation [21, 37, 38]. In South Africa, while few studies have examined ACEs among local populations, the few that exist have nonetheless found high rates of ACEs [39, 40]. For instance, Manyema et al. (2019) studied ACEs in a cohort of children in Soweto, South Africa and found that 35% had four or more ACEs. In addition, in a sample of 223 women in KwaZulu-Natal province, ACEs were significantly associated with substance use and adverse pregnancy outcomes [41]. Moreover, using data from a prospective longitudinal study of adolescents in South Africa, Cluver and colleagues (2015) found a strong relationship between ACEs and mental health problems as well as increased suicidality [42]. Taken together, our results complement the existing literature highlighting the negative impacts of ACEs on mental health symptoms. However, given the hypothesized cumulative association between the ACEs and traumatic events on mental health outcomes, it is critical to assess ACEs in adult outpatient care settings.

Similar to ACEs, the experience of traumatic events during adulthood is associated with a range of adverse physical and mental health outcomes [8]. Our findings validate existing literature in South Africa and elsewhere. Studies have documented the long-term impact of abuse and neglect on mental health, including anxiety, depression and PTSD [43, 44]. Recent studies in South Africa have also highlighted high rates of interpersonal violence and sexual abuse, both of which were found to be common adult traumatic experiences [45, 46]. Interpersonal violence and assault are highly prevalent in South Africa and strongly correlated with psychological sequelae. Specifically, one study found that 31% of the participants were victims of recurrent assault injury and community violence, all of which have been found to exacerbate mental health problems [47]. Other studies have noted the relationship between trauma and historical legacies associated with violence from the apartheid period [48]. In another study of women living with HIV in South Africa, 51% reported sexual violence and 75% reported physical violence from an intimate partner [49]. Research has also highlighted a significant increase in gender-based violence (GBV) in recent years, dramatically impacting women’s and children’s mental health [50, 51]. Several studies have highlighted how GBV has been a longstanding problem in South Africa [52, 53]. Consistent with other studies, our results found females reported significantly higher levels of depression and anxiety compared to males [55–56].

South Africa has seen increasing rates of depression and anxiety in recent years, particularly in the context of the COVID-19 pandemic [32, 57, 58]. Notably, in 2020, the rising COVID-19 infection rates and declining human mobility likely contributed to the increased frequency of major depressive and anxiety disorders. Specifically, COVID-19 in South Africa induced lockdowns, stay-at-home directives, reduced use of public transportation, business and school closures, and a decline in social connections, all of which have been shown to be associated with poor mental health outcomes [59, 60]. For a population already burdened with multiple stressors and mental health challenges, a combination of COVID-19 control measures potentially exacerbated mental health issues for vulnerable groups [61–63]. Studies estimate that between 2.2 and 2.8 million South Africans lost their jobs due to the first COVID-19 lockdown. Another 40% remained unemployed even after the lockdown was lifted, and the economy experienced a 5% decline in GDP during this period [64, 65]. At the time this study was conducted, the economy had still yet to recover fully. Evidence also suggests that individuals who have experienced ACEs are more vulnerable to stress during COVID-19 [48, 66]. As participants in this study lived primarily in townships characterized by high poverty levels, crime, violence, and HIV and AIDS, other studies have also established these as risk factors for mental illness [45, 67].

This study found higher rates of depression, stress, and anxiety among younger participants as well as those with higher education. Findings from other studies in Mozambique [68], Nigeria [69]. and Vietnam [70] support the association between higher education and anxiety and/or depressive disorders. These studies posit that increased health literacy among younger, educated populations may reflect a shift away from traditional or supernatural conceptualizations leading them to be more likely to present at biomedical services for care thus resulting in this relationship [69]. This study’s authors examined questions related to pathways to care and traditional healing in a separate analysis [71]. Although the results of our study are associative, unemployment and lack of opportunities to earn money are probably mediating factors in the relationship between education and depression. Researchers have highlighted how two-thirds of South Africans 16 to 34 years have never worked, and that although South Africa is considered a middle-income country, there is a considerable lack of job opportunities among youths [72]. Therefore, having a university or secondary level education without a job may have contributed to the symptoms of anxiety and depression, particularly among younger participants. Not surprisingly, South Africa’s unemployment rate is nearly 30% and likely higher in the townships. Hence, it is likely that a person with high educational attainment expects to get a good job and live a better life. However, they may lack the social and structural opportunity to capitalize on their education given the relatively difficult economic conditions in South Africa.

Lastly, it is important to note that this study found that participants’ self-rated better physical health was associated with lower anxiety and depressive disorder levels. Mental and physical health have a bi-directional relationship [73]. This relationship is mediated by several lifestyle behaviors, including physical activity, social interaction, alcohol, and past mental and physical health [73]. For instance, better physical health likely improves physical activity, which in turn has a positive association with better mental health. Consistent with other studies [74, 75], it is critical to develop and strengthen these mechanisms among individuals receiving psychiatric care earlier to build resilience, lessen the impact of risk factors associated with depression and anxiety, and facilitate a smooth recovery with evidence-based trauma-informed psychosocial support – a form of treatment currently lacking in public psychiatric facilities in South Africa.

These findings should be interpreted in light of the following limitations. The data in this study are cross-sectional; therefore, we cannot establish causality with the estimated associations. Additionally, the use of screening scales to measure mental health (compared with structured clinical interviews) tends to overestimate rates of psychiatric illness [76, 77]. Ultimately, our findings highlight the need for sustained engagement to support patients in care and promote positive mental health outcomes.

## Conclusions

Given high exposure to ACEs and trauma and the associated impact on the mental health of individuals, families, and communities, there is a need to strengthen and scale innovative combination interventions that holistically address the needs of people in low-resource settings. These findings underscore the need for additional screening of ACEs and trauma, and providing holistic treatment services that address multiple stressors originating from different points in the patient’s life course. Mental health care provision in South Africa should provide more psychosocial care for psychiatric patients, as currently the majority of patients receiving treatment at public facilities receive little to no counseling or therapy due to monetary constraints of providing this care. Additional research could examine ways to provide cost-effective forms of psychosocial support in these facilities through task shifting models which employ lay counselors, for example. Furthermore, interventions that reduce or mitigate social, familial, economic, and environmental adversities may be protective against depression and anxiety [4, 78, 79].

### Electronic supplementary material

Below is the link to the electronic supplementary material.

Supplementary Material 1: Correlates of Depression, Anxiety and Stress Symptom Severity, Stratified by Sex (N=309)

## Data Availability

The datasets used and/or analyzed during the current study are available from the corresponding author upon reasonable request.
