# Supplementary material for: Adverse childhood experiences, traumatic events, and mental health among adults at two outpatient psychiatric facilities in Johannesburg, South Africa: a cross-sectional analysis
Source: BMC Psychiatry. 2023 Aug 10;23:581. doi: 10.1186/s12888-023-05085-0 (PMC10413614; doi:10.1186/s12888-023-05085-0)
Supplement: Supplementary file 1 — Supplementary Material 1: Correlates of Depression, Anxiety and Stress Symptom Severity, Stratified by Sex (N=309) [file 12888_2023_5085_MOESM1_ESM.docx]

| **Supplementary Table 1: Correlates of Depression Symptom Severity, Stratified by Sex (N=309)** | | | | |
| --- | --- | --- | --- | --- |
|  | Adjusted unstandardized regression coefficient (95% CI), p-value  **Women (N=172)** | | Adjusted unstandardized regression coefficient (95% CI), p-value  **Men (N=137)** | |
|  | **Model 1** | **Model 2** | **Model 3** | **Model 4** |
| **Variable** |  |  |  |  |
| ACEs, per point | 0.40 (-0.01, 0.80), 0.06 |  | **0.60 (0.18, 1.03), 0.006** |  |
| Traumatic events, per point |  | **0.82 (0.34, 1.31), 0.001** |  | **0.75 (0.24, 1.27), 0.005** |
| *Footnote: The model adjusted for age, per year; history of psychiatric illness in the family; people in the household, per person; children in the family, per child; monthly income, per ZAR; marital status; education; self-rated physical health, per point; and HIV status.* | | | | |

| **Supplementary Table 2: Correlates of Anxiety Symptom Severity, Stratified by Sex (N=309)** | | | | |
| --- | --- | --- | --- | --- |
|  | Adjusted unstandardized regression coefficient (95% CI), p-value  **Women (N=172)** | | Adjusted unstandardized regression coefficient (95% CI), p-value  **Men (N=137)** | |
|  | **Model 1** | **Model 2** | **Model 3** | **Model 4** |
| **Variable** |  |  |  |  |
| ACEs, per point | **0.45 (0.15, 0.75), 0.003** |  | **0.55 (0.20, 0.90), 0.002** |  |
| Traumatic events, per point |  | **0.67 (0.31, 1.03), P<.001** |  | **0.47 (0.03, 0.91), 0.04** |
| *Footnote: The model adjusted for age, per year; history of psychiatric illness in the family; people in the household, per person; children in the family, per child; monthly income, per ZAR; marital status; education; self-rated physical health, per point; and HIV status.* | | | | |

| **Supplementary Table 3: Correlates of Stress Symptom Severity, Stratified by Sex (N=309)** | | | | |
| --- | --- | --- | --- | --- |
|  | Adjusted unstandardized regression coefficient (95% CI), p-value  **Women (N=172)** | | Adjusted unstandardized regression coefficient (95% CI), p-value  **Men (N=137)** | |
|  | **Model 1** | **Model 2** | **Model 3** | **Model 4** |
| **Variable** |  |  |  |  |
| ACEs, per point | 0.31 (-0.11, 0.74), 0.15 |  | **1.30 (0.81, 1.78), P<.001** |  |
| Traumatic events, per point |  | **0.93 (0.42, 1.44), P<.001** |  | **1.14 (0.52, 1.77), P<.001** |
| *Footnote: The model adjusted for age, per year; history of psychiatric illness in the family; people in the household, per person; children in the family, per child; monthly income, per ZAR; marital status; education; self-rated physical health, per point; and HIV status.* | | | | |

| **Supplementary Table 4: Correlates of Depression Symptom Severity (N=309)** | | |
| --- | --- | --- |
|  | Standardized coefficient (95% confidence intervals), p values | Standardized coefficient (95% confidence intervals), p values |
| **Variable** | **Model 1** | **Model 2** |
| ACEs, per point | **0.19 (0.08, 0.29), 0.001** |  |
| Traumatic events, per point |  | **0.23 (0.13, 0.33), <.001** |
| Age, per year | **-0.15 (-0.27, -0.03), 0.013** | **-0.21 (-0.33, -0.10), <.001** |
| Sex | **-1.15 (-0.25, 0.04), 0.008** | **-0.18 (-0.28, -0,07), 0.001** |
| History of psychiatric illness in the family | 0.05 (-0.06, 0.15), 0.392 | 0.05 (-0.05, 0.15), 0.287 |
| People in the household, per person | -0.09 (-0.419, 0.01), 0.074 | **-0.10 (-0.20, -0.01), 0.037** |
| Children in the family, per child | -0.07 (-0.18, 0.05), 0.265 | -0.06 (-0.17, 0.06), 0.333 |
| Monthly income, per ZAR | -0.05 (-0.15, 0.06), 0.384 | -0.04 (-0.14, 0.06), 0.452 |
| Marital Status | 0.04 (-0.06, 0.14), 0.399 | 0.03 (-0.07, 0.13), 0.601 |
| Education | **0.12 (0.01, 0.22), 0.031** | 0.10 (0.00, 0.20), 0.060 |
| Self-rated physical health, per point | **-0.36 (-0.47, -0.26), <.001** | **-0.35 (-0.45, -0.25), <.001** |
| HIV positive | -0.06 [-0.16, 0.04], 0.277 | -0.06 (-0.16, 0.03), 0.200 |
|  |  |  |

| **Supplementary Table 5: Correlates of Anxiety Symptom Severity (N=309)** | | |
| --- | --- | --- |
|  | Standardized coefficient (95% confidence intervals), p values | Standardized coefficient (95% confidence intervals), p values |
| **Variable** | **Model 1** | **Model 2** |
| ACEs, per point | **0.25 (0.14, 0.35), <.001** |  |
| Traumatic events, per point |  | **0.22 (0.12, 0.32), <.001** |
| Age, per year | **-0.13 (-0.25, -0.01), 0.031** | **-0.20 (-0.32, -0.08), .001** |
| Sex | -0.10 (-0.21, 0.01), 0.064 | **-0.14 (-0.25, -0.03), 0.011** |
| History of psychiatric illness in the family | 0.07 (-0.03, 0.18), 0.163 | **0.10 (0.00, 0.20), 0.045** |
| People in the household, per person | -0.03 (-0.13, 0.06), 0.488 | -0.05 (-0.15, 0.05), 0.300 |
| Children in the family, per child | -0.08 (-0.19, 0.04), 0.210 | -0.06 (-0.18, 0.05), 0.282 |
| Monthly income, per ZAR | -0.04 (-0.14, 0.06), 0.450 | -0.03 (-0.13, 0.07), 0.580 |
| Marital Status | 0.03 (-0.07, 0.13), 0.541 | 0.01 (-0.09, 0.11), 0.820 |
| Education | **0.13 (0.02, 0.24), 0.016** | 0.11 (0.00, 0.21), 0.045 |
| Self-rated physical health, per point | **-0.33 (-0.43, 0.23), <.001** | **-0.32 (-0.43, 0.22), <.001** |
| HIV positive | -0.06 (-0.16, 0.04), 0.217 | -0.07 (-0.18, 0.03), 0.152 |

| **Supplementary Table 6: Correlates of Stress Symptom Severity (N=309)** | | |
| --- | --- | --- |
|  | Standardized coefficient (95% confidence intervals), p values | Standardized coefficient (95% confidence intervals), p values |
| **Variable** | **Model 1** | **Model 2** |
| ACEs, per point | **0.27 (0.16, 0.38), <.001** |  |
| Traumatic events, per point |  | **0.27 (0.17, 0.37), <.001** |
| Age, per year | -0.08 (-0.21, 0.04), 0.172 | **-0.17 (-0.29, -0.05), 0.007** |
| Sex | -0.09 (-0.20, 0.02), 0.112 | **-0.13 (-0.24, -0.02), 0.019** |
| History of psychiatric illness in the family | 0.01 (-0.10, 0.12), 0.849 | 0.04 (-0.07, 0.14), 0.489 |
| People in the household, per person | -0.03 (-0.14, 0.07), 0.508 | -0.05 (-0.15, 0.05), 0.302 |
| Children in the family, per child | -0.00 (-0.13, 0.12), 0.957 | 0.01 (-0.11, 0.13), 0.887 |
| Monthly income, per ZAR | -0.04 (-0.14, 0.07), 0.498 | -0.03 (-0.13, 0.08), 0.631 |
| Marital Status | 0.10 (-0.01, 0.20), 0.067 | 0.08 (-0.03, 0.18), 0.155 |
| Education | 0.07 (-0.04, 0.18), 0.217 | 0.04 (-0.06, 0.15), 0.422 |
| Self-rated physical health, per point | **-30 (-.041, -0.19), <.001** | **-0.29 (-0.39, -0.18), <.001** |
| HIV positive | 0.04 (-0.06, 0.15), 0.422 | 0.03 (-0.07, 0.13), 0.561 |
